# Induction of metastatic potential by TrkB via activation of IL6/JAK2/STAT3 and PI3K/AKT signaling in breast cancer

**DOI:** 10.18632/oncotarget.5522

**Published:** 2015-10-20

**Authors:** Min Soo Kim, Won Sung Lee, Joon Jeong, Seong-Jin Kim, Wook Jin

**Affiliations:** ^1^ Laboratory of Molecular Disease and Cell Regulation, Department of Molecular Medicine, School of Medicine, Gachon University, Incheon 406-840, Korea; ^2^ Department of Surgery, Kangnam Severance Hospital, Yonsei University, Kangnam, Seoul 146-92, Korea; ^3^ CHA Cancer Institute, CHA University, Seongnam-si, Kyunggi-do 463-400, Korea; ^4^ Gachon Medical Research Institute, Gil Medical Center, Gachon University, Incheon 405-760, Korea

**Keywords:** TrkB, epithelial-mesenchymal transition (EMT), IL-6/JAK2/STAT3 pathway, PI3K/AKT pathway, metastasis and tumorigenicity

## Abstract

In metastatic breast cancers, the acquisition of metastatic ability, which leads to clinically incurable disease and poor survival, has been associated with acquisition of epithelial-mesenchymal transition (EMT) program and self-renewing trait (CSCs) via activation of PI3K/AKT and IL6/JAK2/STAT3 signaling pathways. We found that TrkB is a key regulator of PI3K/AKT and JAK/STAT signal pathway-mediated tumor metastasis and EMT program. Here, we demonstrated that TrkB activates AKT by directly binding to c-Src, leading to increased proliferation. Also, TrkB increases Twist-1 and Twist-2 expression through activation of JAK2/STAT3 by inducing c-Src-JAK2 complex formation. Furthermore, TrkB in the absence of c-Src binds directly to JAK2 and inhibits SOCS3-mediated JAK2 degradation, resulting in increased total JAK2 and STAT3 levels, which subsequently leads to JAK2/STAT3 activation and Twist-1 upregulation. Additionally, activation of the JAK2/STAT3 pathway via induction of IL-6 secretion by TrkB enables induction of activation of the EMT program via induction of STAT3 nuclear translocation. These observations suggest that TrkB is a promising target for future intervention strategies to prevent tumor metastasis, EMT program and self-renewing trait in breast cancer.

## INTRODUCTION

There are several discrete steps in the biological cascade of metastasis, loss of cellular adhesion, increased motility and invasiveness, entry and survival into circulation, exit into new tissue, and eventual colonization of a distant site [1, 2]. During cancer pathogenesis, a transdifferentiation program defined as epithelial–mesenchymal transition (EMT) is activated to acquire the ability to execute the multiple steps of the invasion-metastasis cascade [3–5]. Furthermore, certain epithelial cells that pass through an EMT acquire the self-renewing traits associated with normal tissue (SCs) and cancer stem cells (CSCs) [6, 7].

Metastatic breast cancers are more chemoresistant forms of breast cancer with expression of high levels of EMT markers and exhibit a CD44^high^/CD24^low^ antigenic phenotype, which results in their having stem cell-like characteristics (CSCs). There is accumulating evidences of a role of PI3K/AKT and JAK/STAT3 signaling pathways in tumor metastasis and EMT. Activation of the PI3K/AKT pathway, IL-6/JAK2/STAT3 pathway, and stem cell-like characteristics contribute to the poor outcomes of metastatic breast cancers [8, 9]. Moreover, activated JAK2/STAT3 increases AKT activation through the induction of AKT [10], while increased STAT3 and AKT activation leads to tumor development and EMT [11, 12].

Independent of these findings, TrkB, a member of the tropomyosin-related kinase (Trk) family of neurotrophin receptors, is critical to biological processes in the developing and mature nervous systems, including neuronal growth, differentiation, and survival [13]. Also, TrkB is overexpressed in various cancer types, including prostate cancer [14], multiple myeloma [15], Wilms’ tumor [16], lung cancer [17], pancreatic cancer [18] and neuroblastoma [19], and is associated with poor prognosis [20, 21]. In addition, TrkB expression is important to the regulation of angiogenesis [22] and acts as a potent mediator of anoikis resistance in epithelial cells through AKT activation [23, 24]. However, the signaling mechanisms of TrkB-mediated PI3K/AKT activation have remained unclear. Thus, even though TrkB expression may play an important role in the tumorignicity of many tumors, it is currently unclear how TrkB regulate interactions between metastasis and EMT at the invasive front of the tumor. In this report, we identified a new network involved in tumor metastasis and EMT that regulates and coordinates with TrkB. Surprisingly, we found that TrkB is primarily present in human breast cancer and acts as a key regulator of the PI3K/AKT and JAK/STAT signal pathway-mediated tumor metastasis, EMT, and self-renewing trait.

## RESULTS

### c-Src is required for TrkB-induced activation of the PI3K-AKT pathway

It is reported that ectopic TrkB expression in RIE-1 cells suppresses anoikis through AKT activation [25]. However, the signaling mechanisms that activates PI3K/AKT signaling pathway have remained unclear. In addition, c-Src appears to be important for multiple aspects of tumor progression, including proliferation, disruption of cell/cell contacts, migration, invasiveness, resistance to apoptosis, and angiogenesis [26]. c-Src is also known to activate PI3K/AKT and the MAP/extracellular signal-regulated kinase (ERK) kinase (MEK)/ERK cascades through both focal adhesion kinase (FAK)-dependent and FAK-independent pathways [27]. This raises the possibility that TrkB regulates tumor progression by activation of PI3K/AKT signaling pathway via regulation of c-Src. However, the correlation of TrkB and c-Src in breast cancer has remained unknown and none of these findings indicates a link between these two sets of phenomena.

**Figure 1:**
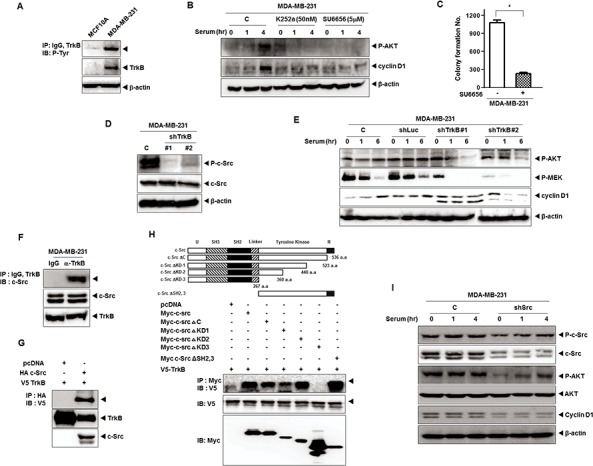
TrkB-mediated c-Src activation induces activation of the PI3K/AKT and Ras-MAPK pathway **A.** Identification of TrkB expression and autophosphorylation in MDA-MB-231 cell lines. Cell extracts were immunoprecipitated using anti-TrkB antibody and Gamma-bind beads, after which they were immunoblotted with anti-phosphotyrosine antibody. **B.** Western blot analysis of expression of phospho-AKT and cyclin D1 in MDA-MB-231 cells treated with 50 nM K252a or 5 μM SU6656. β-actin was used as a loading control. **C.** Colony formation assay of MDA-MB-231 cells treated with 5 μM SU6656 (*n* = 3). *P < 0.001, *t*-test. **D.** Western blot analysis of expression of phospho-c-Src and c-Src in MDA-MB-231 control-shRNA or TrkB-shRNA cells. β-actin was used as a loading control. **E.** Western blot analysis of expression of phospho-AKT, phospho-MEK, and cyclin D1 in MDA-MB-231 control-shRNA or TrkB-shRNA cells. β-actin was used as a loading control. **F.** Identification of complex formation of endogenous TrkB/c-Src in MBA-MB-231 cells. **G.** TrkB interacts with the c-Src after transfection with c-Src or TrkB. **H.** Identification of the c-Src region responsible for TrkB interaction. Immunoblot analysis of whole-cell lysates and immunoprecipitates derived from 293T cells transfected with the V5-TrkB and Myc-c-Src deletion constructs was conducted as indicated. **I.** Western blot analysis of expression of phospho-c-Src, phospho-AKT, c-Src, AKT, and cyclin D1 in MDA-MB-231 control-shRNA or c-Src-shRNA cells. β-actin was used as a loading control.

We initially examined whether the elevated levels of TrkB associated with the increase in phosphorylation of TrkB. As shown in Figure 1A, a highly metastatic MDA-MB-231 human breast cancer cell line expresses high level of TrkB and it is phosphorylated. Therefore, we investigated whether TrkB collaborated with c-Src in tumorigenicity of breast cancer. We found that pretreatment with K252a (an inhibitor of TrkC tyrosine kinases) and SU6656 (an inhibitor of c-Src tyrosine kinases) markedly reduced levels of phosphorylated AKT, as well as induction of cyclin D1, indicating that the PI3K/AKT pathway, and cyclin D1 act downstream of c-Src in MDA-MB-231 cells (Figure 1B). We also investigated whether the inhibition of c-Src kinase activity blocks the colony formation of MDA-MB-231 cells using SU6656. Upon addition of 5 μM SU6656, the colony formation of MDA-MB-231 cells was markedly suppressed as indicated by failure to form microscopic or macroscopic colonies in soft agar when scored after 16 days (Figure 1C).

These observations led us to speculate that TrkB might modulate expression or activation of c-Src. To determine whether TrkB contributes to regulation of c-Src in MAD-MB-231 cells, we selected highly metastatic MDA-MB-231 and Hs578T cells stably expressing the TrkB-shRNAs. TrkB-shRNA partially suppressed the expression of endogenous TrkB (i.e., a 80% reduction) in MDA-MB-231 and Hs578T cells (Supplementary Figure 1A). We next examined the expression of Src in MDA-MB-231 control-shRNA or TrkB-shRNA cells. c-Src expression was not affected by the knockdown of TrkB in MDA-MB-231 cells, but c-Src phosphorylation levels were significantly decreased in MDA-MB-231 TrkB-shRNA cells, suggesting that TrkB activates c-Src in these cells (Figure 1D). We further examined whether TrkB expression regulates MEK1/2 and AKT activation, as well as the induction of cyclin D1 expression. Levels of phosphorylated (activated) MEK1/2 and AKT as well as cyclin D1 expression were markedly reduced in MDA-MB-231 TrkB-shRNA cells relative to MDA-MB-231 control-shRNA cells (Figure 1E). These results suggest that TrkB affects proliferation of MDA-MB-231 cells through activation of c-Src.

To further investigate the induction of c-Src phosphorylation by TrkB, we examined whether TrkB directly interacts with c-Src in MDA-MB-231 cells. Interestingly, endogenous TrkB was able to interact with endogenous c-Src under physiological conditions (Figure 1F). To confirm this finding in a transient transfection system, V5-tagged TrkB and HA-tagged c-Src were transfected into 293T cells. As shown in Figure 1G, TrkB interacted strongly with c-Src. To identify the functional domain of c-Src responsible for the interaction with TrkB, we used a series of c-Src deletion constructs. The c-Src mutant lacking the N-terminal domain (c-Src ΔKD2, 3), c-Src ΔC, c-Src ΔKD-1, and c-Src ΔKD-2 still interacted with TrkB. However, the deletion of amino acid residues 275–360 a.a. (c-Src ΔKD-3) abrogated the TrkB interaction (Figure 1H). These results clearly indicate that the region including amino acids 275–360 of c-Src, which includes the tyrosine kinase domain, is required for TrkB interaction.

Therefore, we examined whether c-Src activation is involved in the TrkB-induced Mek1 and AKT activation as well as cyclin D1 expression. To test this notion, we selected MDA-MB-231 cells stably expressing the c-Src-shRNAs. As shown in Supplementary Figure 1B, knockdown of c-Src in MDA-MB-231 cells (MDA-MB-231 c-Src-shRNA cells) resulted in decreases in the expression of endogenous c-Src of over 60%. Moreover, TrkB-induced AKT activation and cyclin D1 expression were significantly reduced in c-Src-shRNA cells (Figure 1I), indicating that TrkB might be involved in tumorigenicity and metastasis of breast cancer through TrkB-mediated activation of PI3K/AKT and Ras-MAPK pathways or upregulation of cyclin D1 expression. Taken together, our findings show that c-Src is essential for the ability of TrkB to constitutively activate the Ras-MAPK and the PI3K-AKT pathways and to induce cyclin D1 overexpression.

#### TrkB-induced c-Src activation is involved in JAK2 activation, but not with STAT3 activation

**Figure 2:**
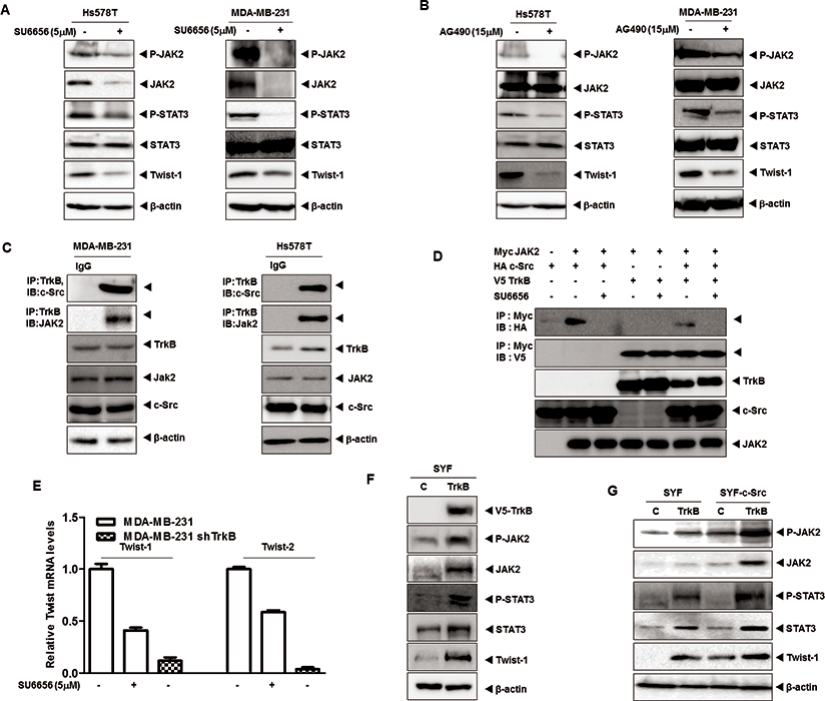
TrkB led to Twist-1 upregulation through activation of the JAK/STAT3 pathway after TrkB/c-Src/JAK2 complex formation **A.** Western blot analysis of expression of phospho-JAK2, JAK2, phospho-STAT3, STAT3, and Twist-1 proteins in Hs578T and MDA-MB-231 cells treated with 5 μM SU6656. β-actin was used as a loading control. **B.** Western blot analysis of expression of phospho-JAK2, JAK2, phospho-STAT3, STAT3, and Twist-1 proteins in Hs578T and MDA-MB-231 cells treated with 15 μM AG490. β-actin was used as a loading control. **C.** Identification of complex formation of endogenous TrkB/c-Src/JAK2 in MBA-MB-231 and Hs578T cells. **D.** Western blot analysis of whole-cell lysates and immunoprecipitates derived from 293T cells transfected with V5-TrkB, HA-c-Src and Myc-Jak2 constructs as indicated with or without 5 μM SU6656. **E.** The relative expression levels of mRNA encoding Twist-1 and Twist-2 in MDA-MB-231 cells stably expressing control shRNA or TrkB-shRNA treated with or without 5 μM SU6656, as determined by quantitative RT-PCR. **F.** Western blot analysis of the expression of phospho-JAK2, JAK2, phospho-STAT3, STAT3, and Twist-1 in SYF cells expressing either pCAG or pCAG/TrkB. β-actin was used as a loading control. **G.** Western blot analysis of expression of phospho-JAK2, JAK2, phospho-STAT3, STAT3, and Twist-1 in SYF cells or SYF-c-Src cells expressing either pCAG or pCAG/TrkB. β-actin was used as a loading control.

Activation of STAT3 is required for c-Src-mediated proliferation, survival and transformation [28–31], and also mediates resistance to trastuzumab [32]. Moreover, activated STAT3 leads to AKT activation [10] and upregulates Twist-1, an EMT-induced transcription factor [33, 34]. These observations led us to speculate that TrkB is involved in regulation of STAT3 activation and PI3K/AKT signaling through activation of c-Src, and that it is functionally linked to regulation of Twist-1. To test the effect of TrkB and c-Src on STAT3 activation, we initially examined whether inhibition of c-Src activity in the Hs578T and MDA-MB-231 cells affected STAT3 activation. We found that JAK2 and STAT3 activation, as well as Twist-1 expression were markedly decreased after treatment of Hs578T and MDA-MB-231 cells with SU6656. Interestingly, JAK2 expression was markedly decreased; whereas STAT3 expression was not affected by SU6656 treatment (Figure 2A). Moreover, the inhibition of JAK2 activation in Hs578T and MDA-MB-231 cells significantly reduced JAK and STAT3 phosphorylation and Twist-1 expression after treatment with the JAK2 inhibitor, AG490. However, JAK2 and STAT3 expression levels were not significantly different after treatment with AG490 (Figure 2B). Our results suggest that c-Src activation is involved in the upregulation of JAK2, which is correlated with JAK2 phosphorylation, but not with STAT3 activation.

Given-mentioned role of TrkB as c-Src activator via direct interaction, it is possible that the TrkB/c-Src complex could activate JAK2 via interaction. Indeed, we found that both TrkB and c-Src interacted with JAK2 after transient transfection (Supplementary Figure 2A). Moreover, endogenous TrkB interacted with endogenous c-Src/JAK2 in MDA-MB-231 and Hs578T cells (Figure 2C). Furthermore, JAK2 interacted with TrkB even in the absence of c-Src or SU6656 treatment, indicating a direct binding of TrkB to JAK2 (Figures 2D and Supplementary Figure 2B). To identify the JAK2 functional domain responsible for its interaction with TrkB, we used a series of JAK2 deletion constructs. A JAK2 mutant containing the tyrosine kinase domain interacted with TrkB, whereas TrkB did not interact with the JAK2 B41, SH2, or pseudokinase domain mutants (Supplementary Figure 2C). To further determine whether TrkB-JAK2 formed a complex in the absence of c-Src, we expressed TrkB in SYF cells derived from triple knock-out mouse embryos lacking members of the Src kinase family (c-Src, Yes, and Fyn) (Klinghoffer et al., 1999). We transiently transfected SYF-TrkB and SYF-c-Src-TrkB cells with JAK2. As shown in Supplementary Figure 2D, TrkB-JAK2 complexes were detected in both c-Src-deficient SYF-TrkB and SYF-c-Src-TrkB cells. Moreover, TrkB in the absence of c-Src was sufficient to induce of JAK2 expression in SYF-TrkB cells (Supplementary Figure 2E).

Interestingly, MDA-MB-231 TrkB-shRNA cells demonstrated much lower Twist-1 and Twist-2 mRNA levels than MDA-MB-231 cells treated with SU6656 (Figure 2E). Additionally, TrkB knockdown exhibited an almost 3-fold decrease in promoter activity of Twist-1 and Twist-2 genes relative to SU6656 treatment (Figures 2E and Supplementary 2F). These results suggest that Twist-1 or Twist-2 upregulation by TrkB might be regulated via at least two downstream pathways involving the c-Src. Moreover, TrkB-mediated JAK2 and STAT3 phosphorylation levels were markedly increased in SYF-TrkB and SYF-cSrc-TrkB cells. Furthermore, JAK2, STAT3, and Twist-1 expression was markedly increased in SYF-TrkB or SYF-cSrc-TrkB cells compared to SYF or SYF-c-Src cells (Figure 2F and 2G). These results were consistent with Twist-1 and Twist-2 luciferase activity in SYF-TrkB and SYF-c-Src-TrkB cells. Also, ectopic TrkB expression increased promoter activity of Twist-1 and Twist-2 genes in SYF cells and SYF-c-Src cells. Moreover, both TrkB and c-Src synergistically increased Twist-1 and Twist-2 luciferase activity (Supplementary Figure 2G), and significantly increased JAK2 and STAT3 expression, suggesting that TrkB directly activates JAK2 and STAT3 through upregulation of JAK2/STAT3 expression with or without c-Src.

#### TrkB induces JAK2 stabilization through inhibition of its ubiquitination

**Figure 3:**
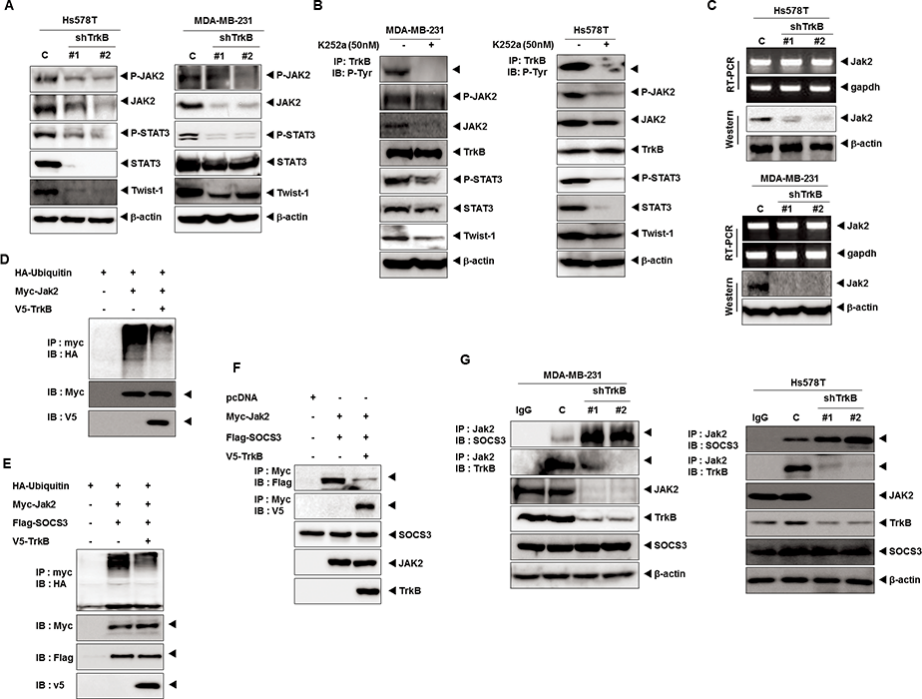
TrkB upregulates JAK2 expression by inhibiting the SOCS3-mediated degradation of JAK2 **A.** Western blot analysis of expression of phospho-JAK2, JAK2, phospho-STAT3, STAT3, and Twist-1 proteins in Hs578T and MDA-MB-231 control-shRNA or TrkB-shRNA cells. β-actin was used as a loading control. **B.** Tyrosine kinase activity of TrkB is important to induction of the JAK2/STAT3 pathway. Western blot analysis of expression of phospho-JAK2, JAK2, phospho-STAT3, STAT3, and Twist-1 proteins in Hs578T and MDA-MB-231 cells treated with 50 nM K252a. **C.** The relative expression of JAK2 in Hs578T and MDA-MB-231 control-shRNA or TrkB-shRNA cells, as determined by RT-PCR or western blotting. GAPDH and β-actin were used as loading controls. **D.** Western blot analysis of whole-cell lysates and immunoprecipitates derived from 293T cells transfected with the V5-TrkB, HA-Ubiquitin and Myc-Jak2 constructs, as indicated. **E.** Western blot analysis of whole-cell lysates and immunoprecipitates derived from 293T cells transfected with the V5-TrkB, HA-Ubiquitin, Flag-SOCS3 and Myc-Jak2 constructs, as indicated. **F.** Western blot analysis of whole-cell lysates and immunoprecipitates derived from 293T cells transfected with the V5-TrkB, Flag-SOCS3, or Myc-Jak2 constructs as indicated. **G.** Endogenous TrkB inhibits SOCS3/JAK2 complex formation. The cell lysates were subjected to immunoprecipitation using anti-IgG and anti-JAK2 Abs followed by immunoblotting with anti-SOCS3, anti-JAK2, and anti-TrkB antibodies.

We next attempted to investigate whether TrkB knockdown, which was identified above as being activated by the JAK2/STAT3/Twist-1 axis without c-Src, affects activation of the JAK2/STAT3/Twist-1 axis in Hs578T and MDA-MB-231 cells. Immunoblotting analysis revealed that JAK2 and STAT3 phosphorylation levels were markedly reduced in Hs578T and MDA-MB-231 TrkB-shRNA cells relative to its control-shRNA cells. Interestingly, the JAK2, STAT3, and Twist expression levels were also significantly reduced in Hs578T and MDA-MB-231 TrkB-shRNA cells (Figure 3A). Additionally, STAT3, Twist-1 and Twist-2 mRNA expression was markedly reduced in response to TrkB knockdown (Supplementary Figure 3A and 3C). Moreover, promoter activity of STAT3, Twist-1 and Twist-2 genes was significantly decreased in Hs578T and MDA-MB-231 TrkB-shRNA cells compared to its control-shRNA cells (Supplementary Figure 3B and 3D). Furthermore, the inhibition of TrkB activation by K252a treatment significantly reduced JAK2 and STAT3 phosphorylation and downregulated JAK2, STAT3, and Twist-1 expression (Figure 3B). These observations suggest that tyrosine kinase activity of TrkB is required for JAK2/STAT3/Twist-1 induction as well as JAK2/STAT3 expression.

Because TrkB knockdown reduced the JAK2 protein levels, we initially focused on JAK2 expression. TrkB knockdown significantly reduced the JAK2 protein levels, but had no effect on JAK2 mRNA levels (Figure 3C), suggesting that TrkB post-translationally controls JAK2 protein stability. The SOCS3 protein is known to bind to JAK2 and suppress JAK2 activity through direct binding to the JAK2 catalytic center and promotion of the proteasomal degradation of JAK2 [35]. Therefore, we investigated whether JAK2 stabilization by TrkB was mediated through the suppression of JAK2 ubiquitination. When JAK2 was immunoprecipitated from the cell lysate, level of JAK2 ubiquitination was markedly reduced when TrkB was coexpressed (Figure 3D). TrkB also suppressed JAK2 ubiquitination, even in the presence of SOCS3, which mediated the negative feedback inhibition of the JAK–STAT pathway (Figure 3E). Therefore, we investigated whether TrkB induced JAK2 activation by blocking the ability of SOCS3 to ubiquitinate and degrade JAK2 through SOCS3-JAK2 complex formation. As expected, TrkB coexpression significantly reduced the level of SOCS3-associated JAK2 (Figure 3F). We next compared the JAK2-SOCS3 complex formation in MDA-MB-231 and Hs578T TrkB-shRNA cells to that of the control-shRNA cells. The level of endogenous SOCS3 associated with JAK2 was dramatically increased in MDA-MB-231 and Hs578T TrkB-shRNA cells, which was correlated with decreased JAK2 (Figure 3G). These results indicated that TrkB inhibited proteasome-mediated JAK2 degradation by blocking SOCS3-JAK2 complex formation, leading to activation of the JAK2/STAT3/Twist-1 axis.

### Increased secretion of IL-6 protein by TrkB increases the nuclear translocation of STAT3

**Figure 4:**
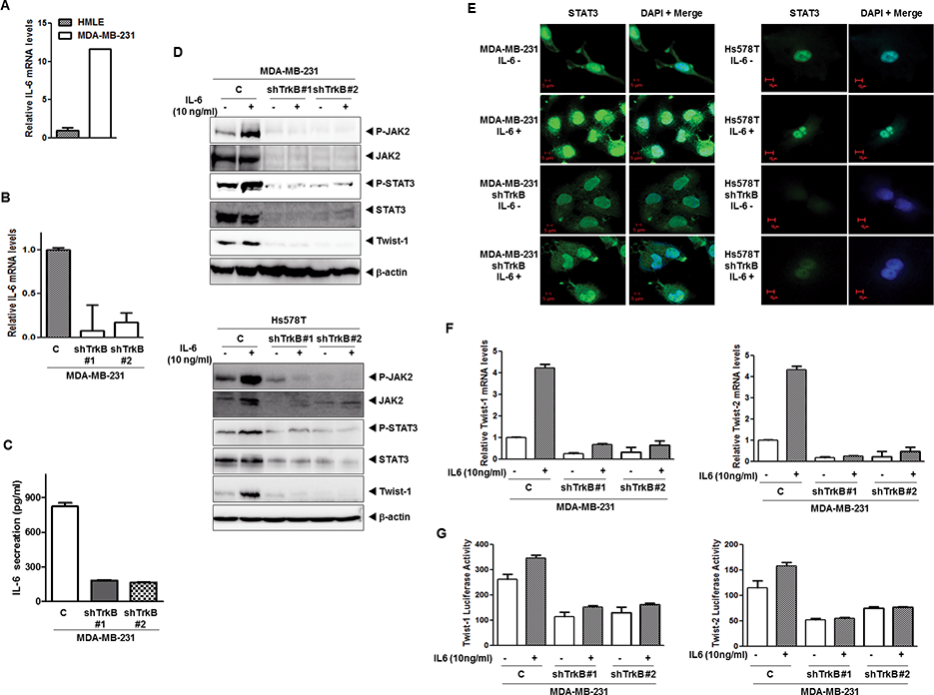
Induction of IL-6 secretion by TrkB enhances nuclear translocation of STAT3 and upregulates Twist-1 and Twist-2 **A.** The relative expression of mRNA encoding IL-6 in MDA-MB-231 cells relative to that of HMLE cells, as determined by quantitative RT-PCR. The 18S mRNA level was used to normalize the variability in template loading. **B.** The relative expression of the mRNA encoding IL-6 in MDA-MB-231 control-shRNA or TrkB-shRNA cells, as determined by quantitative RT-PCR. The 18S mRNA level was used to normalize the variability in template loading. **C.** IL-6 secretion by MDA-MB-231 control-shRNA or TrkB-shRNA cells, as determined by ELISA. The data are reported as the means ± SEM. **D.** Western blot analysis of the expression of phospho-JAK2, JAK2, phospho-STAT3, STAT3, and Twist-1 proteins in MDA-MB-231 and Hs578T control-shRNA or TrkB-shRNA cells treated with or without IL-6. β-actin was used as a loading control. **E.** Immunofluorescence images of STAT3 in MDA-MB-231 and Hs578T control-shRNA or TrkB-shRNA cells treated with IL-6. The green signal represents staining of the corresponding protein, while the blue signal represents DAPI staining. **F.** The relative expression levels of mRNA encoding Twist-1 and Twist-2 in MDA-MB-231 control-shRNA or TrkB-shRNA cells treated with IL-6, as determined by quantitative RT-PCR. The 18S mRNA level was used to normalize the variability in template loading. **G.** Promoter activity of Twist-1 and Twist-2 genes in MDA-MB-231 control-shRNA or TrkB-shRNA cells treated with or without IL-6. Each bar represents the mean ± SEM of three experiments.

STAT3 phosphorylation by IL-6 promotes its nuclear translocation and DNA binding followed by the transactivation of genes involved in a number of cellular functions, including proliferation, differentiation, and survival (Catlett-Falcone et al., 1999; Heinrich et al., 2003; Oshiro et al., 2001; Puthier et al., 1999). Moreover, the IL-6/STAT3/JAK2 pathway is involved in the maintenance of stem cell–like cancer cells [36] because CD44^+^CD24^+^ and CD44^+^CD24^−^ breast cancer cells have high phospho-STAT3 via their expression of genes such as *IL6*, *PTGIS*, and *HAS1*, which activate an autocrine loop [8]. Furthermore, high IL-6 levels have been associated with poor clinical outcome in breast cancer patients [37]. We speculated that STAT3 activation by TrkB depends on the activation of autocrine signaling loops. To investigate whether TrkB is relevant to IL-6 expression, we examined IL-6 expression in a panel of HMLE and MDA-MB-231 or its TrkB knockdown cells. The mRNA levels of IL-6 were significantly upregulated in MDA-MB-231 cells relative to HMLE and its TrkB knockdown cells (Figure 4A and 4B). Together, increased secretion of IL-6 protein (4.5- to 5-fold) by MDA-MB-231 cells, relative to TrkB knockdown cells, correlated with increased mRNA levels of IL-6 (Figure 4C).

To further examine the TrkB-mediated regulation of the JAK2/STAT3 pathway, we assessed IL-6-induced JAK2 and STAT3 activation in MBA-MB-231 and Hs578T control-shRNA or TrkB-shRNA cells. Relative to the MDA-MB-231 and Hs578T control-shRNA cells, TrkB-shRNA cells showed significantly reduced activation and expression of JAK2/STAT3, as well as Twist-1 expression after stimulation with or without IL-6 (Figure 4D). Furthermore, STAT3 nuclear translocation after IL-6 treatment was markedly increased in MDA-MB-231 and Hs578T control-shRNA cells relative to TrkB knockdown cells (Figure 4E). Additionally, the expression and promoter activity of Twist-1 and Twist-2 genes was markedly increased in MDA-MB-231 and Hs578T control-shRNA cells, but not in its TrkB-shRNA cells (Figure 4F and 4G). Together, these observations indicate that the role of TrkB in activation of the IL6/JAK2/STAT3 pathway can also serve to induce entry into a stem cell-like state, allowing the derivation of cells with metastatic potential.

### TrkB induces EMT through activation of the JAK2/STAT3 pathway and PI3K/AKT pathway

**Figure 5:**
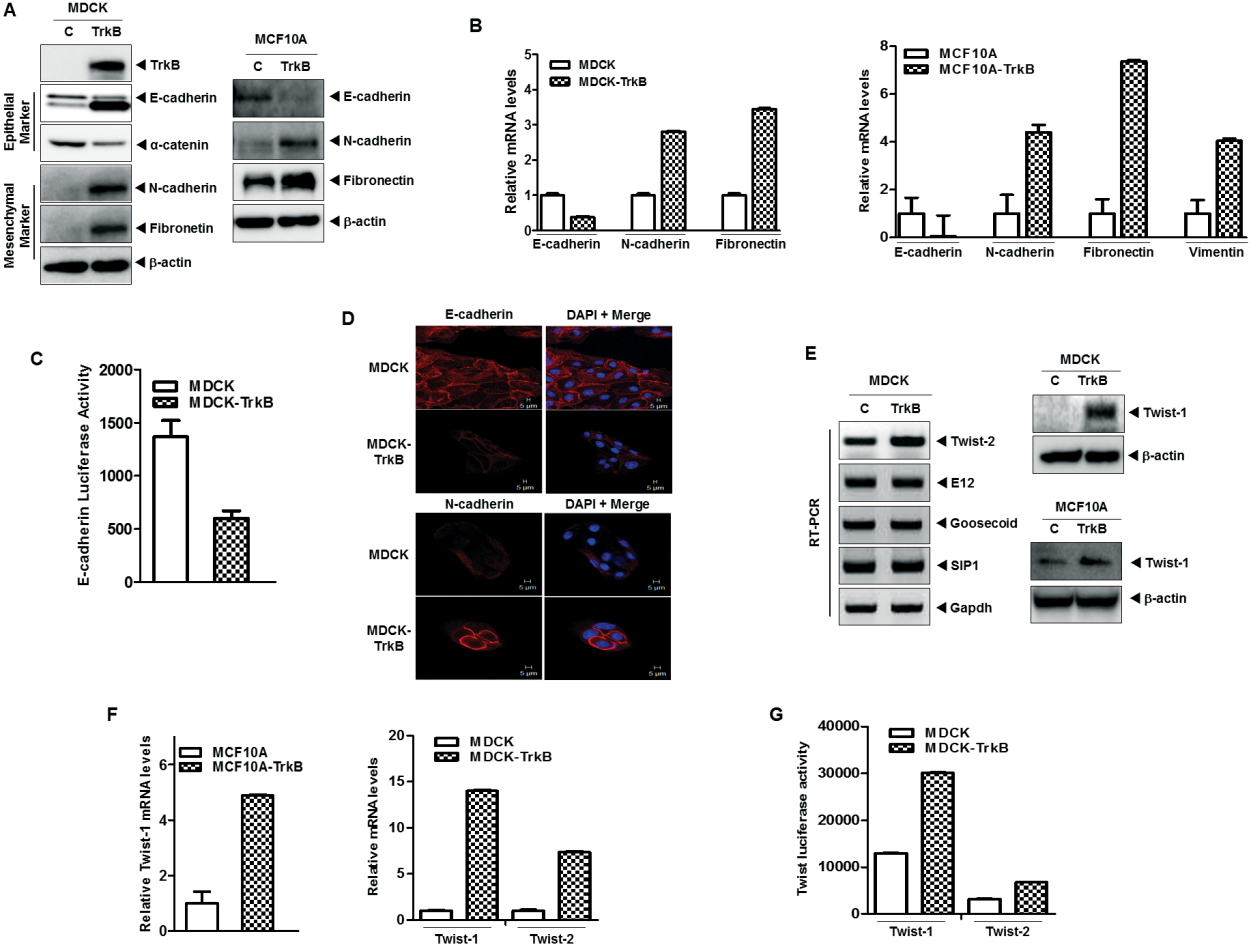
TrkB induces EMT program via upregulation of Twist-1 and Twist-2 expression **A.** Western blot analysis of the expression of TrkB, E-cadherin, α-catenin, N-cadherin, and fibronetin proteins in MDCK, MCF10A, MDCK-TrkB, and MCF10A-TrkB cells. β-actin was used as a loading control. **B.** The relative expression levels of mRNA encoding E-cadherin, N-cadherin, vimentin, and fibronectin in MDCK, MCF10A, MDCK-TrkB, and MCF10A-TrkB cells, as determined by quantitative RT-PCR. The 18S mRNA level was used to normalize the variability in template loading. **C.** Promoter activity of E-cadherin gene in MDCK and MDCK-TrkB cells. Each bar represents the mean ± SEM of three experiments. **D.** Immunofluorescence images of E-cadherin and N-cadherin in MDCK, and MDCK-TrkB. The red signal represents staining of the corresponding protein, while the blue signal represents DAPI staining. **E.** Western blot analysis of the expression of Twist-1 protein (right) and RT-PCR analysis (left) of mRNA encoding Twist-2, E12, Goosecoid, SIP1, and Slug in MDCK, MCF10A, MDCK-TrkB, and MCF10A-TrkB cells. **F.** The relative expression levels of mRNA encoding Twist-1 and Twist-2 in MDCK, MCF10A, MCF10A-TrkB and MDCK-TrkB cells, as determined by quantitative RT-PCR. The 18S mRNA level was used to normalize the variability in template loading. **G.** Promoter activity of Twist-1 and Twist-2 genes in MDCK and MDCK-TrkB cells.

The results of a recent study suggests that Twist-1 and Twist-2 induce activation of the EMT program to maintain the resulting mesenchymal state [38–40]. Moreover, cooperation between the Stat3 and AKT signaling pathways results in tumor development and EMT in the prostates of mice [11]. Therefore, we investigated whether the contribution of TrkB to tumor metastasis involved EMT induction through activation of the JAK2/STAT3 and PI3K/AKT pathways. To address this possibility, we examined whether expression of TrkB was able to promote an EMT in normal mammalian cells. To accomplish this, we overexpressed TrkB in MDCK and MCF10A cells by ectopic expression of TrkB. As anticipated, MDCK-TrkB and MCF10A-TrkB cells downregulated the expression of mRNA and proteins of epithelial markers (such as E-cadherin, and α-catenin), while they upregulated the expression of mRNA and proteins of mesenchymal markers (such as N-cadherin, fibronectin, and vimentin) (Figure 5A and 5B). Also, promoter activity of E-cadherin gene was also efficiently suppressed in the MDCK-TrkB cells compared to the MDCK-control cells (Figure 5C). In addition, immunostaining revealed that, relative to the expression of E-cadherin in MDCK-TrkB cells, the level of E-cadherin in MDCK cells was strongly induced and N-cadherin expression was markedly reduced (Figure 5D).

During the process of tumor metastasis, which is often enabled by EMT program, pleiotropically acting transcription factors, such as E12, Goosecoid, SIP1, Snail, Twist-1, and Twist-2, are induced that orchestrate EMT programs [6, 41]. These recent study and our above observations led us to speculate that TrkB might contribute to induction of EMT via modulation of pleiotropically acting transcription factors. We examined whether TrkB regulates the expression of SIP1, E12, Goosecoid, Twist-1, and Twist-2. There was a considerable increase in the expression of EMT-inducing transcription factors, specifically Twist-1, and Twist-2, in MDCK-TrkB and MCF10A-TrkB cells but not with SIP1, E12, and Goosecoid expression (Figure 5E), which was correlated with increased luciferase activity and expression of mRNA encoding Twist-1 and Twist-2 (Figure 5F and 5G).

Also, relative to the parental MDA-MB-231 control-shRNA cells, TrkB-shRNA cells significantly downregulated the expression of mesenchymal markers such as N-cadherin and fibronectin, whereas they upregulated the expression of epithelial markers (such as E-cadherin and α-catenin). Moreover, the levels of Twist-1 was significantly downregulated in MDA-MB-231 TrkB-shRNA cells relative to MDA-MB-231 control-shRNA cells (Supplementary Figure 4A). Furthermore, we found that the immunoactivity of epithelial or mesenchymal markers in MDA-MB-231 control-shRNA cells was correlated with the expression levels of MDCK-TrkB cells as shown in Supplementary Figure 4B. In addition, immunostaining data revealed that the Twist-1 protein in MDA-MB-231 control-shRNA cells was strongly upregulated (Supplementary Figure 4C) relative to the expression of Twist-1 in MDA-MB-231 TrkB-shRNA cells, which was correlated with the expression of mRNA encoding Twist-1 and Twist-2. These results provided further evidence that TrkB contributed to the EMT phenotype through activation of the PI3K/AKT and IL6/JAK2/STAT3 pathway.

### Knockdown of TrkB significantly reduces tumor growth and metastasis *in vivo*

**Figure 6:**
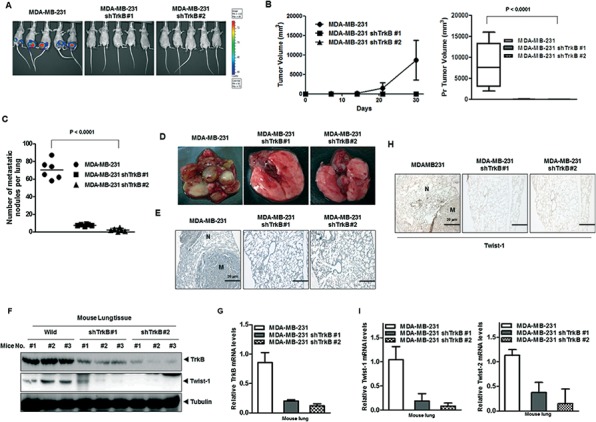
Suppression of TrkB expression inhibited metastasis from the mammary gland to the lung **A, B.** Tumor formation by MDA-MB-231 control-shRNA or MDA-MB-231 TrkB-shRNA cells. 1.0 × 10^5^ cells were implanted in the mammary fat pads of mice (*n* = 7 mice/group). A *P* < 0.0001 was considered to indicate significance for ANOVA. **C.** Lung metastasis by MDA-MB-231 control-shRNA or MDA-MB-231 TrkB-shRNA cells. The total numbers of lung metastatic nodules in each mouse in each group were counted using a dissection scope (*n* = 6 mice/group). A *P* < 0.0001 was considered to indicate significance for ANOVA. **D.** Representative images of lungs from mice harboring MDA-MB-231 control-shRNA or TrkB-shRNA cell mammary tumors for 30 days after implantation of tail veins of mice. **E.** Representative H&E staining in sections of the lungs from Figure 6D. N, Normal lung tissue; M, metastatic nodule. **F, G.** Western blot analysis (F) and quantitative RT-PCR (G) of TrkB, and Twist-1 in tumor cells recovered from the lungs of individual mice expressing either MDA-MB-231 control-shRNA or MDA-MB-231 TrkB-shRNA. Tubulin and 18S mRNA were used as loading controls. **H.** Representative immunohistochemical images of Twist-1 staining in sections of the lungs from individual mice expressing either MDA-MB-231 control-shRNA or MDA-MB-231 TrkB-shRNA (magnification: 200×). **I.** The expression levels of the mRNA encoding Twist-1 and Twist-2 in tumor cells recovered from the lungs of individual mice expressing either MDA-MB-231 control-shRNA or TrkB-shRNA cells.

To determine the contribution of TrkB to primary tumor formation, we injected MDA-MB-231 cells expressing either TrkB-shRNA or control-shRNA into the mouse mammary fat pads of BALB/c Nu/Nu mice and examined the resulting primary tumors 30 days later. Control MDA-MB-231 cells formed primary mammary tumors at identical rates, whereas primary tumor formation by MDA-MB-231 TrkB-shRNA cells was markedly decreased (Figure 6A and 6B). These results demonstrate that TrkB is essential to primary tumor formation by MDA-MB-231 cells.

To determine if the loss of TrkB expression affected the ability of MDA-MB-231 cells to metastasize, MDA-MB-231 cells expressing either TrkB-shRNA or the control shRNA were injected into the tail veins of BALB/c Nu/Nu mice, and their lungs were examined for metastases 35 days after injection. Suppression of TrkB expression strongly reduced the number of metastatic nodules relative to MDA-MB-231 control-shRNA cells (Figure 6C and 6D). Additionally, histological analyses confirmed that the number of micrometastatic lesions was drastically reduced in the lungs of mice with MDA-MB-231 TrkB-shRNA cells (Figure 6E). Importantly, few metastatic nodules in the lungs of mice carrying TrkB-shRNA cells retained TrkB expression, but TrkB expression in the lungs of mice with TrkB-shRNA cells was greatly reduced relative to the lungs of mice carrying control-shRNA cells (Figure 6F and 6G). Moreover, quantitative RT-PCR and immunohistochemistry analysis of Twist-1 and Twist-2 revealed reduced expression in the lungs of mice injected with MDA-MB-231 TrkB-shRNA cells compared to their control counterparts (Figure 6H and 6I). These results indicated that expression of TrkB is required for the full metastatic ability of highly metastatic MDA-MB-231 breast cancer cells.

## DISCUSSION

TrkB is overexpressed in several human cancers, ranging from neuroblastomas to pancreatic ductal adenocarcinomas, and its overexpression suppresses anoikis as an EMT inducer by regulation of Zeb1 [23, 42]. Independent of these discoveries, CD44^high^/CD24^low^ cells, which show a stem cell-like phenotype, are enriched in highly metastatic breast cancer cells (basal or claudin-low breast subtypes) have been proposed to be resistant to cancer therapies through activation of the PI3K/AKT pathway, IL-6/JAK2/STAT3 pathway, and EMT [8, 43–47]. Although increasing evidence implies that altered PI3K/AKT signaling in response to ectopic TrkB promotes tumor formation and metastasis, the molecular mechanisms of TrkB-mediated PI3K/AKT modulation in breast cancer have remained unknown, and none of the findings reported to date hinted at a link between these two sets of phenomena.

In the present study, we found that activation of the MEK and PI3K/AKT pathway through TrkB-mediated c-Src activation after TrkB-c-Src complex formation induced tumorigenicity and metastatic potential of breast cancer. These our results are supported by those of a previous study in which the phosphatidylinositol-3-OH kinase/protein kinase B signaling pathways required for EMT, anokis suppression, c-Src activated PI3K/AKT and Ras/MAPK cascades [23, 27, 48, 49].

We further dissected the role of TrkB in tumorigenicity and metastasis. Activated STAT3 was able to transform cells *in vitro* and was required for cell transformation of a number of oncogenes and activation of STAT3 by interleukin-6 or expression of activated c-Src induced Twist expression at the protein and mRNA levels [29–31, 34, 50–52]. These previous observations led us to investigate whether TrkB regulates STAT3 activation via c-Src activation. We found that c-Src activation by TrkB was required for JAK2 activation through interaction with JAK2, but not with STAT3. TrkB significantly upregulated the JAK2 protein level, which had no effect on the JAK2 mRNA level. Moreover, TrkB in the absence of c-Src is sufficient to activate JAK2/STAT3 through blocking of JAK2 degradation by SOCS3 after directly binding to the JAK2, as well as upregulation of EMT related transcription factors, such as Twist-1 and Twist-2. A great deal of research has described the role of SOCS3, which specifically prevents activation of STAT3 by IL-6 [35, 53–57]. Our studies further uncovered TrkB as a key regulator in coordinating the actions of JAK2 and c-Src in tumorigenesis.

Recent studies showed that the IL-6 inflammatory feedback loop leads to CSC self-renewal and induction of EMT, both of which are implicated in tumor metastasis and poor outcomes by therapeutic resistance [8, 9, 36, 37, 58]. Moreover, IL-6 secretion induced by HER2 overexpression elicited JAK2/STAT3 activation [59]. Therefore, we investigated whether TrkB enforces an autocrine loop of IL-6/JAK2/STAT3 via induction of IL-6 secretion. Although IL-6 is regulated by multiple factors, increased secretion of IL-6 protein (4.5- to 5-fold) by TrkB was found to be correlated with increased mRNA levels of IL-6. Furthermore, induction of STAT3 nuclear translocation by TrkB induced EMT via increased expression of EMT related transcription factors such as Twist-1 and Twist-2.

Recent evidence indicates transcription factors Twist-1 and Twist-2, which are master regulators of embryonic morphogenesis, play an essential role in metastasis, CSCs and EMT of breast cancer [39, 40, 60–66]. Both proteins override oncogene induced premature senescence by abrogating key regulators of the p53- and Rb-dependent pathways. Moreover, AKT2 is a transcriptional regulatory target of Twist that acts downstream of Twist to promote cancer cell survival, migration, and invasion [67]. In addition, JAK2/STAT3 activity is required for activation of the PI3K/AKT pathway via upregulation of AKT1 promoter activity [10, 68]. Those studies and our results presented herein indicate that downstream mediation of TrkB is more complex, and is likely to be cellular context dependent and/or promoter dependent. Although the results of this study by no means exclude the involvement of other factors, they do suggest that activation of the IL-6 autocrine loop by TrkB maintains the metastatic potential and CSCs self-renewal via activation of the JAK2/STAT3 pathway, PI3K/AKT pathway, and EMT (Supplementary Figure 5). Overall, we identified a new molecular and functional network present in cancer metastasis that regulates and coordinates with TrkB. Moreover, we demonstrated that TrkB has the potential for use as a new target for improving the treatment efficacy of metastatic breast cancer.

## MATERIALS AND METHODS

### Cell culture and reagents

Human breast cancer (MCF10A, SUM149, MDA-MB-231, and Hs578T), SYF, 293T, and MDCK cell lines were maintained as previously described [40, 69, 70]. The protein kinase inhibitor K252a and SU6656, and AG490 was purchased from Calbiochem.

### Plasmids

Each of the two shRNA-encoding oligonucleotides against mouse and human TrkB was designed and verified to be specific to TrkB through BLAST searches against the mouse and human genomes, respectively. The primers corresponding to TrkB were cloned into the pLKO lentiviral vector to generate the TrkB-shRNA expression plasmid (Supplementary Table 1). shRNA that did not match any known mouse- or human-coding cDNA was used as a control.

### Antibodies, western blotting, immunoprecipitation, and immunofluorescence

Assays were performed as previously described, with modification [40, 69]. The antibodies were obtained from the following companies: anti-HA(Y-11), anti-c-Src, and anti-Myc (9E10) were from Santa Cruz; anti-V5 was from Invitrogen; anti-TrkB and anti-SOCS3 were from Abcam; anti-phospho-c-Src, anti-Twist-1, anti-STAT3, anti-phospho-STAT3, anti-JAK2, and anti-phospho-JAK2 were from Cell Signaling Technology; and anti-E-cadherin, anti-fibronectin, anti-N-cadherin, anti-α-catenin, and anti-β-catenin were from BD Transduction.

### Viral production, colony formation assay, and RT-PCR

All assays were performed as previously described [69, 71]. The primer sequences used to amplify the genes are listed in the Supplementary Tables (Supplementary Table 2).

### Quantitative RT-PCR

The primer sequences are listed in the supplemental experimental procedures (Supplementary Table 2). Total RNA was isolated using RNeasy Mini Kits (Qiagen) according to the manufacturer's instructions and reverse transcribed with the hexa-nucleotide mix (Roche). The resulting cDNA was employed in PCR using SYBR-Green Master PCR mix and Taqman master PCR mix (Applied Biosystems). All PCR analyses were conducted in triplicate using the 7900HT Fast Real-Time PCR System (Applied Biosystems). All quantitations were normalized to endogenous control 18S RNA. Specific TrkB (Hs00178811_m1) and 18S (Hs99999901_s1) quantitative probes for Taqman RT-PCR were obtained from Applied Biosystems and the primer sequences used to amplify the genes are listed in Supplementary Table 2.

### Luciferase reporter assay

Cells that were 50% confluent in 12-well dishes were transfected using Lipofectamine 2000 (Invitrogen). A total of 0.5 μg *E-cadherin*, *Twist-1*, *Twist-2*, or *STAT3* reporter gene constructs and 0.5 μg of pCMV-β-gal were cotransfected per well. Cell extracts were prepared 48 hrs after transfection, and the luciferase activity was quantified using an Enhanced Luciferase Assay Kit (Promega). All experiments were performed in triplicate.

### Animal studies

BALB/c Nu/Nu mice were purchased from the Korea Research Institute of Bioscience and Biotechnology (KRIBB, South Korea) and handled in compliance with the IACUC. For tumorigenicity studies, 1 × 10^5^ cells suspended in 50 μl PBS/Matrigel (BD Biosciences) were injected subcutaneously into the left and right hind flank regions under anesthesia. Mice were euthanized at 5 weeks and primary tumors were excised for analysis. For tail-vein injection, 1 × 10^5^ cells suspended in 50 μl PBS were injected into the tail vein of 7-week-old BALB/c Nu/Nu mice.

### *In vivo* bioluminescent imaging

Mice were given intraperitoneal injections of 150 ng/g D-Luciferin (Caliper Life Sciences, cat#12279) and anesthetized with 2.5% isoflurane. At 7–8 min after injection, animals were imaged using the Xenogen Spectrum (IVIS-200) imaging system.

### IL-6 ELISA assay

The equal numbers of MDA-MB-231 control-shRNA or TrkB-shRNA cells were plated and cultured for 3 days. Subsequently, conditioned media from these cell cultures were collected and analyzed by the Human IL-6 Quantikine ELIZA kit (R&D systems) according to the manufacturer's instructions.

### Statistical analysis

Data are expressed as the means ± SEM. Statistical analyses of these data were conducted via a Student's *t* test (two-tailed).

## SUPPLEMENTARY FIGURES AND TABLES


