# Supplementary material for: Induction of metastatic potential by TrkB via activation of IL6/JAK2/STAT3 and PI3K/AKT signaling in breast cancer
Source: Oncotarget. 2015 Oct 20;6(37):40158–71. doi: 10.18632/oncotarget.5522 (PMC4741886; doi:10.18632/oncotarget.5522)
Supplement: Supplementary file 1 [file oncotarget-06-40158-s001.pdf]

SUPPLEMENTARY FIGURES AND TABLES

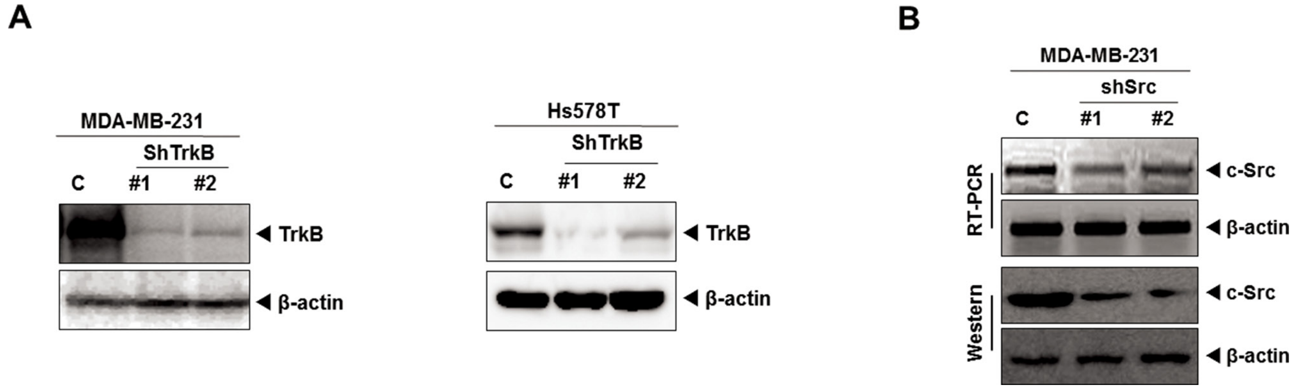

Supplementary Figure 1: Identification of TrkB or c-Src suppression of MDA-MB-231 and Hs578T TrkB-shRNA or c-Src-shRNA cells. A. Western blot analysis of TrkB expression in MDA-MB-231 and Hs578T control-shRNA or TrkB-shRNA cells. **B.** Relative expression of c-Src in MDA-MB-231 control-shRNA or c-Src-shRNA cells, as determined by RT-PCR or western blotting. β-actin were used as loading controls.

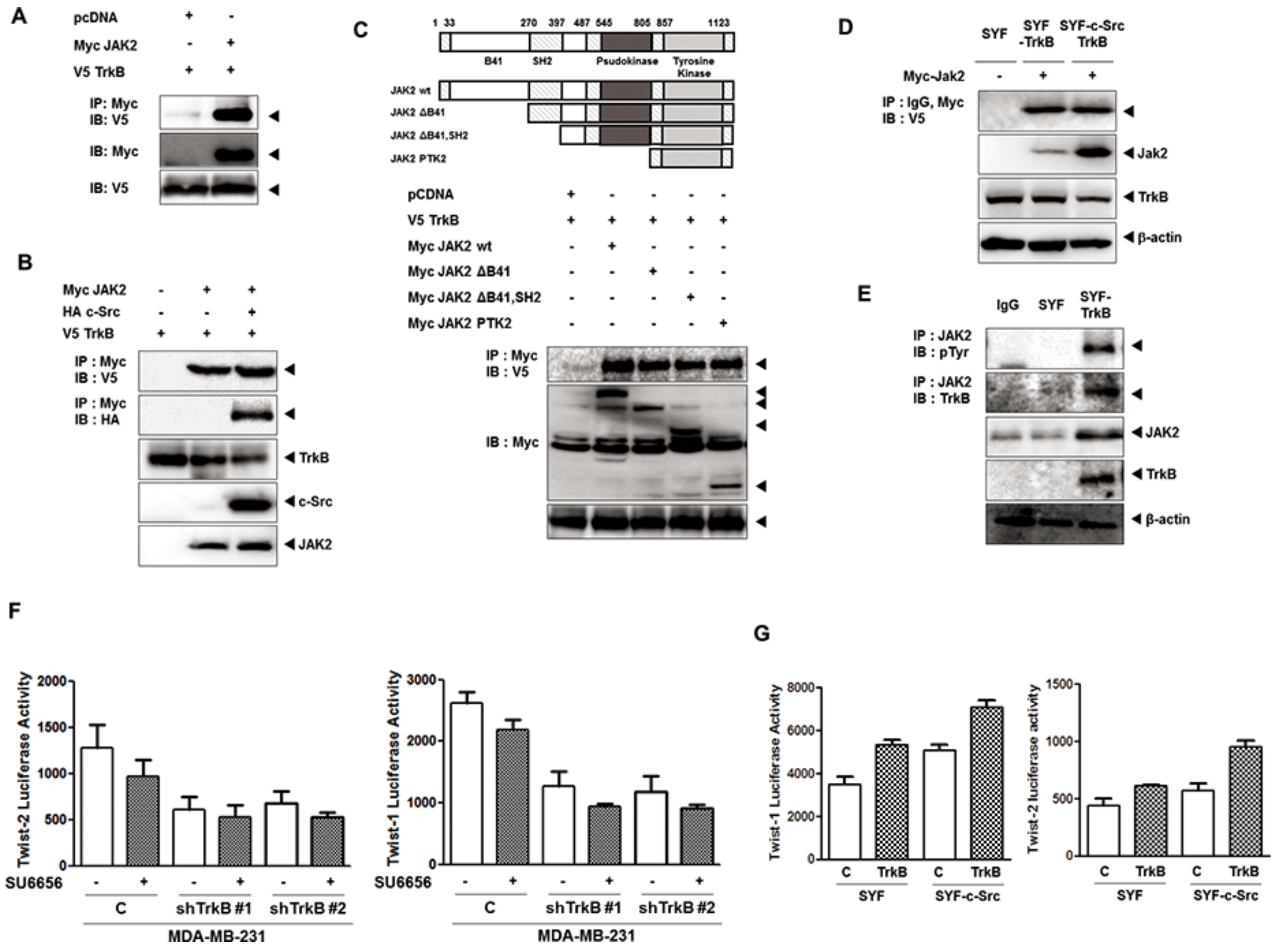

Supplementary Figure 2: TrkB upregulates activation of the JAK2/STAT3 pathway through c-Src-dependent or independent regulation. **A.** Western blot analysis of whole-cell lysates and immunoprecipitates derived from 293T cells transfected with the V5-TrkB and Myc-Jak2 constructs as indicated. **B.** Western blot analysis of whole-cell lysates and immunoprecipitates derived from 293T cells transfected with the V5-TrkB, HA-c-Src, and Myc-Jak2 constructs as indicated. **C.** Identification of JAK2 functional domain responsible for interaction with TrkB. Western blot analysis of whole-cell lysates and immunoprecipitates derived from 293T cells transfected with V5-TrkB, and Myc-Jak2 deletion constructs as indicated. **D.** Western blot analysis of whole-cell lysates and immunoprecipitates derived from SYF-TrkB cells or SYF-c-Src-TrkB cells transfected with the Myc-Jak2 construct as indicated. **E.** Identification of endogenous TrkB/JAK2 complex formation. **F.** Luciferase reporter assay of Twist-1 and Twist-2 in MDA-MB-231 control-shRNA or TrkB-shRNA cells treated with or without SU6656. Each bar represents the mean ± SEM of three experiments. **G.** Luciferase reporter assay of Twist-1 and Twist-2 in SYF, SYF-TrkB, SYF-c-Src, and SYF-c-Src-TrkB cells. Each bar represents the mean ± SEM of three experiments.

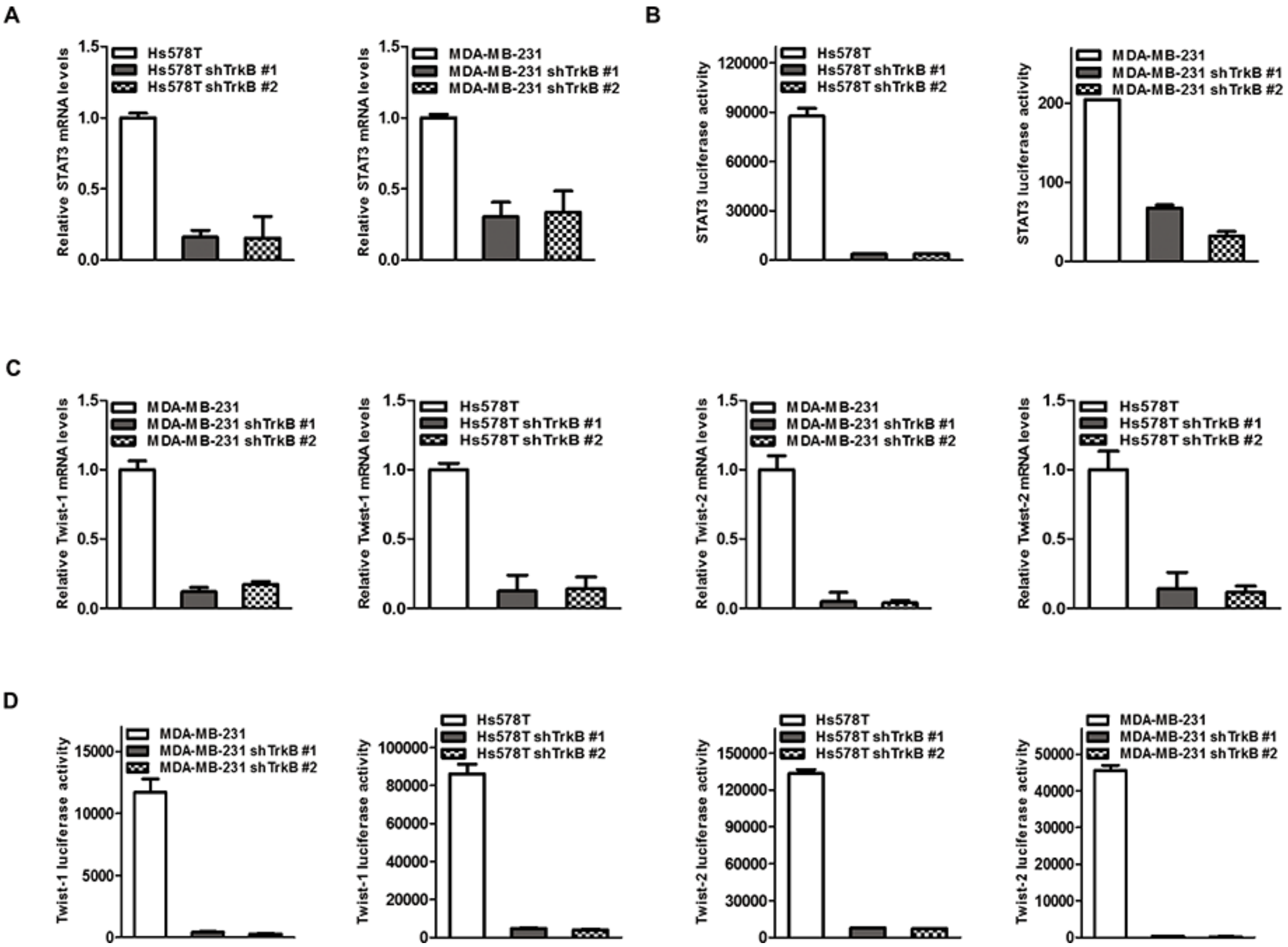

Supplementary Figure 3: TrkB induces expression of Twist-1 and Twist-2 via induction of STAT3 expression. A. Relative expression of mRNA encoding STAT3 in MDA-MB-231 and Hs578T control-shRNA or TrkB-shRNA cells as determined by quantitative RT-PCR. The 18S mRNA level was used to normalize the variability in template loading. **B.** Luciferase reporter assay of STAT3 in MDA-MB-231 and Hs578T control-shRNA or TrkB-shRNA cells. Each bar represents the mean ± SEM of three experiments. **C.** Relative expression of the mRNAs encoding Twist-1 and Twist-2 in MDA-MB-231 and Hs578T control-shRNA or TrkB-shRNA cells as determined by quantitative RT-PCR. The 18S mRNA level was used to normalize the variability in template loading. **D.** Luciferase reporter assay of Twist-1 and Twist-2 in MDA-MB-231 and Hs578T control-shRNA or TrkB-shRNA cells. Each bar represents the mean ± SEM of three experiments.

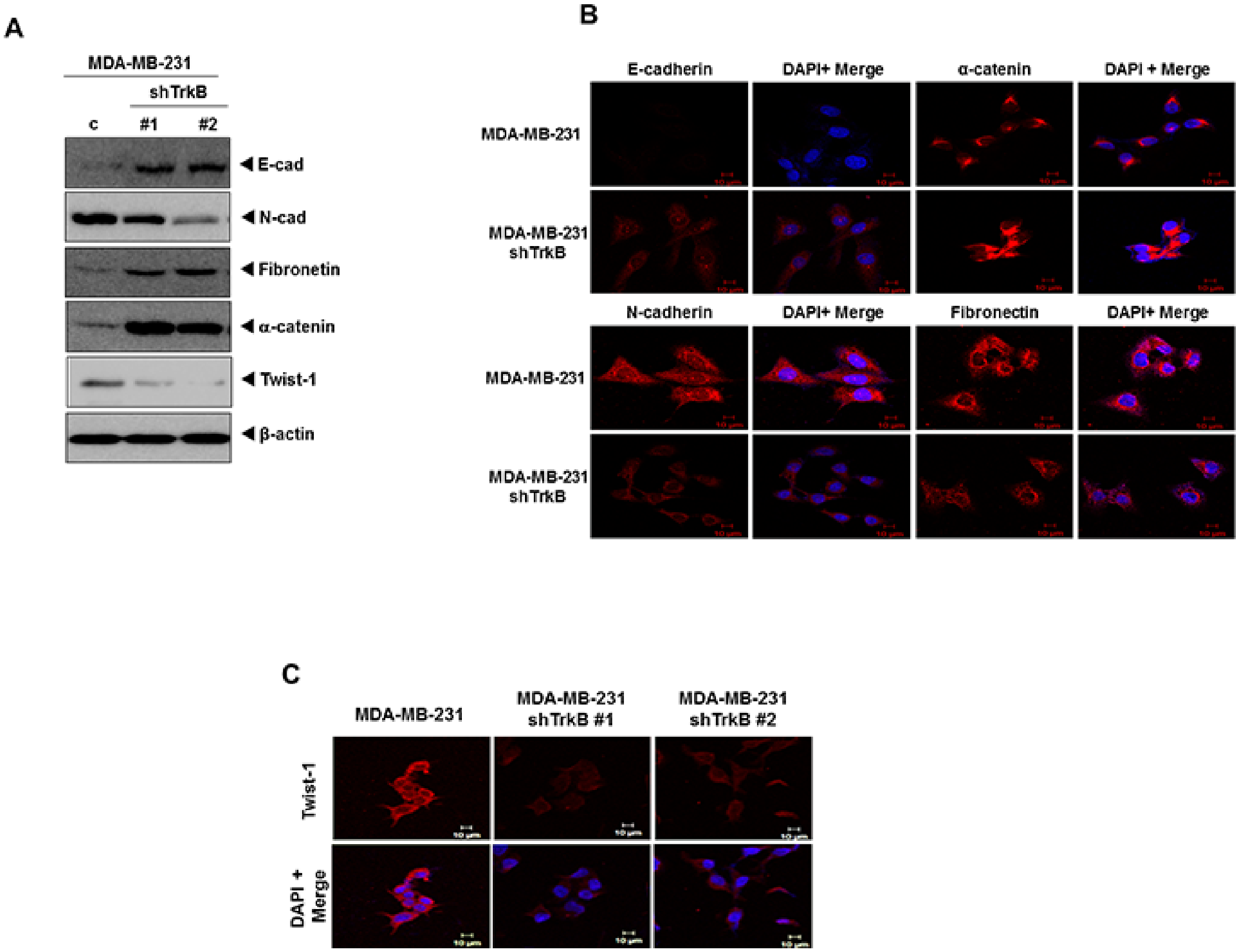

Supplementary Figure 4: TrkB induces EMT program by upregulation of Twist-1. A. Western blot analysis of expression of E-cadherin, N-cadherin, fibronectin, α-catenin, and Twist-1 proteins in MDA-MB-231 control-shRNA or TrkB-shRNA cells. β-actin was used as a loading control. **B.** Immunofluorescence images of E-cadherin, N-cadherin, α-catenin, and fibronectin in MDA-MB-231 control-shRNA or TrkB-shRNA cells. The red signal represents staining of the corresponding protein, while the blue signal represents DAPI staining. **C.** Immunofluorescence images of Twist-1 in MDA-MB-231 control-shRNA or TrkB-shRNA cells. The red signal represents staining of Twist-1 protein, while the blue signal represents DAPI staining.

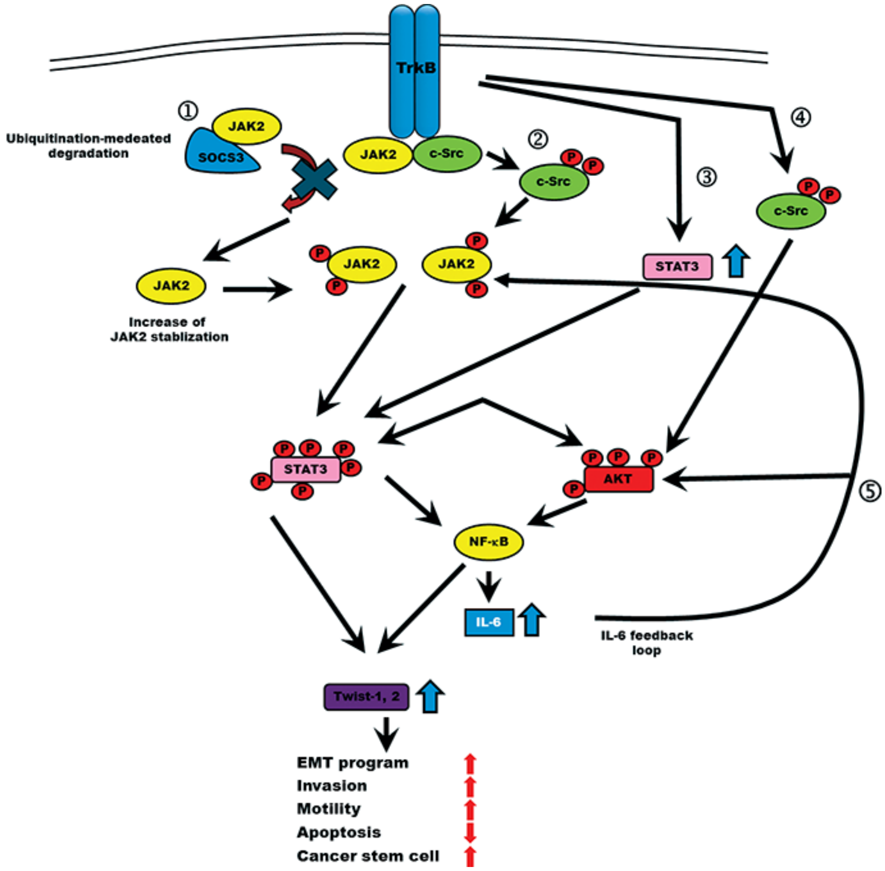

Supplementary Figure 5: Model of activation of PI3K/AKT and IL-6/JAK2/STAT3/Twist-1 pathway by TrkB. TrkB expression increased cell proliferation and motility via (1) upregulation of JAK2 by inhibition of SOCS3-mediated JAK2 degradation and (2) activation of the JAK2/STAT3 pathway through c-Src activation and (3) activation of the JAK2/STAT3/Twist axis through upregulation of STAT3 expression and (4) activation of PI3K/AKT pathway through c-Src activation and (5) positive feedback regulation through induction of IL-6 secretion. Therefore, it is likely that TrkB regulates the activity of the IL-6/JAK2/STAT3/Twist axis to generate relatively unlimited numbers of cancer stem cells, and to obtain the metastatic potential of cancer cells through induction of EMT.

Supplementary Table S1: shRNA sequences for human TrkB genes

Gene name	Primer sequences	
Human TrkB-#1	5′ CCGGCTGGGTGAGGGAGCCTTTGCTCGAG CAAAGGCTCCCTCACCCAGTTTTTG3′	pLKO
Human TrkB-#2	5′ CCGG ATGCTCCACATTGCCAGTCCTCGAG GACTGGCAATGTGGAGCATTTTTG3′	pLKO

Supplementary Table S2: Primer sequences for RT-PCR and quantitative RT-PCR

RT-PCR Primers

Gene	Primers
Human JAK2	F: 5′-CTCGAGGTGCTGAAGCTCCT-3′<br>R: 5′-AAATCATGCCGCCACTGAG-3′
Human Gapdh	F: 5′-CGAGATCCCTCCAAAATCAA-3′<br>R: 5′-TGTGGTCATGAGTCCTTCCA-3′
Dog SIP1	F: 5′-CAGATCGAAGCAGCTCAATG-3′<br>R: 5′-CCTTCAGCGCATAACCTTTC-3′
Dog Goosecoid	F: 5′-GTCAGATCTCCCGCTTTGAG-3′<br>R: 5′-GTCGGGGTACTGGTTCTGC-3′
Dog Slug	F: 5′-TCCATCTGACACCTCATCCA-3′<br>R: 5′-TTGCCACAGATCTTGCAGAC-3′
Dog E12	F: 5′-GCAGACGAAGATGGAAGACC-3′<br>R: 5′-ACCTTCTCCTCCCGATTGAT-3′
Dog Twist-2	F: 5′-GCAAGAAGTCGAGCGAAGAT-3′<br>R: 5′-CTGCAGGACCTGGTGAGGA-3′
Dog gapdh	F: 5′-AAGGTCATCCCTGAGCTGAA-3′<br>R: 5′-AGGCCATGTAGACCATGAGG-3′

Quantitative RT-PCR

Dog E-cadherin	F: 5′-AAAACCCACAGCCTCATGTC-3′<br>R: 5′-TCTAGGGTGGTCACCTGGTC-3′
Dog N-cadherin	F: 5′-CCCAAGACAAGCGACTAAGC-3′<br>R: 5′-TGACAGCTGACCTGAGATGG-3′
Dog Fibronectin	F: 5′-CAGGATGGACATCTGTGGTG-3′<br>R: 5′-GTTGTCTCTCCTGCCCTCAG-3′
Dog gapdh	F: 5′-AACATCATCCCTGCTTCCAC-3′<br>R: 5′-AGACCACCTGGTCCTCAGTG-3′
Human E-cadherin	F: 5′-TGCCCAGAAAATGAAAAAGG-3′<br>R: 5′-GTGTATGTGGCAATGCGTTC-3′
Human N-cadherin	F: 5′-ACAGTGGCCACCTACAAAGG-3′<br>R: 5′-CCGAGATGGGGTTGATAATG-3′
Human Fibronectin	F: 5′-CAGTGGGAGACCTCGAGAAG-3′<br>R: 5′-TCCCTCGGAACATCAGAAAC-3′
Human Vimentin	F: 5′-GAGAACTTTGCCGTTGAAGC-3′<br>R: 5′-GCTTCCTGTAGGTGGCAATC-3′
Human Twist-1	F: 5′-CGACGAGCTGGACTCCAAG-3′<br>R: 5′-CCTCCATCCTCCAGACCGA-3′
Human Twist-2	F: 5′-CAGAGCGACGAGATGGACAA-3′<br>R: 5′-CACACGGAGAAGGCGTAGC-3′
Human STAT3	F: 5′-GGTCTGGCTGGACAATATCATTG-3′<br>R: 5′-ATGATGTACCCTTCGTTCCAAAG-3′
Human 18S	F: 5′-ACCGCAGCTAGGAATAATGGA-3′<br>R: 5′-GCCTCAGTTCCGAAAACCA-3′
